# Chemical Modifications of Laccase from White-Rot Basidiomycete *Cerrena unicolor*

**DOI:** 10.1007/s12010-012-9912-4

**Published:** 2012-10-24

**Authors:** K. H. Kucharzyk, G. Janusz, I. Karczmarczyk, J. Rogalski

**Affiliations:** 1 Department of Biochemistry, Maria Curie-Skłodowska University, Akademicka 19 Street, 20-033 Lublin, Poland; 2 Present Address: Department of Civil & Environmental Engineering, Duke University, 1114 Hudson Hall, Research Drive, Box 90287, Durham, NC 27708-0287 USA

**Keywords:** Laccase, *Cerrena unicolor*, Hydrophobization, Hydrophilization

## Abstract

Laccases belong to the group of phenol oxidizes and constitute one of the most promising classes of enzymes for future use in various fields. For industrial and biotechnological purposes, laccases were among the first enzymes providing larger-scale applications such as removal of polyphenols or conversion of toxic compounds. The wood-degrading basidiomycete *Cerrena unicolor* C-139, reported in this study, is one of the high-laccase producers. In order to facilitate novel and more efficient biocatalytic process applications, there is a need for laccases with improved biochemical properties, such as thermostability or stability in broad ranges of pH. In this work, modifications of laccase isoforms by hydrophobization, hydrophilization, and polymerization were performed. The hydrophobized and hydrophilized enzyme showed enhanced surface activity and higher ranges of pH and temperatures in comparison to its native form. However, performed modifications did not appear to noticeably alter enzyme’s native structure possibly due to the formation of coating by particles of saccharides around the molecule. Additionally, surface charge of modified laccase shifted towards the negative charge for the hydrophobized laccase forms. In all tested modifications, the size exclusion method led to average 80 % inhibition removal for hydrophilized samples after an hour of incubation with fluoride ions. Samples that were hydrophilized with lactose and cellobiose showed an additional 90 % reversibility of inhibition by fluoride ions after an hour of concluding the reaction and 40 % after 24 h. The hydrophobized laccase showed higher level of the reversibility after 1 h (above 80 %) and 24 h (above 70 %) incubation with fluoride ions. The addition of ascorbate to laccase solution before a fluoride spike resulted in more efficient reversibility of fluoride inhibitory effect in comparison to the treatments with reagents used in the reversed sequence.

## Introduction

Laccases (EC 1.10.3.2, *p*-diphenol: oxygen oxidoreductase) are blue copper-containing oxidases, catalyzing the oxidation of *ortho*- and *para*- diphenols, polyphenols, arylamines, aminophenols, and some inorganic ions, while simultaneously reducing molecular dioxygen to water [50, 51, 53]. Laccases are classified into two groups depending on their source: plant and fungal. However, diphenol oxidases have also been identified in bacteria [12] and insects [20]. Three forms of laccase (so called blue, yellow, and white) were discovered in fungi, the most effective producers of this biocatalyst. Laccase is a copper protein containing four metal ions classified as a T1 (one copper), T2 (one copper), and T3 (two coppers) sites according to their spectroscopic characteristics [51]. The T1 copper is responsible for the blue color of the enzyme [48]. An electron from the substrate is transferred to the T1 site (the primary electron acceptor), and then through an intermolecular electron transfer (IET) mechanism via a His-Cys-His bridge to the T2/T3 cluster, where O_2_ is reduced to H_2_O [15, 50].

Recently, *Cerrena unicolor*, was determined as a new fungal source of extracellular laccase, excreting the enzyme under non-induced conditions with the highest activities of 60,000 nkat/l on the 6th day of its cultivation as in Janusz et al. [17]. Several attempts to increase its production including optimization of medium composition and physical parameters of the culture [17, 25, 45] were undertaken. Moreover, laccase from *C. unicolor* was recently purified and characterized as a glycoprotein with a molecular weight of 45 to 75 kDa, depending on the isoform’s composition [2, 33, 45]. Up to date, laccase from *C. unicolor* was used in biodegradation and bioremediation [4, 5], delignification [27], and decolorization [32, 34].

The fact that laccase has a broad specificity for the phenolic substrates makes it an attractive candidate as a component of biosensor [19], for the determination of total phenols [44] and biofuel cell cathodes [20, 23, 38]. It is known that the substrates attach to the binding site of laccase by hydrophobic interactions [44]. Thus, the effectiveness of electrode constructed of laccase would depend on the quality and quantity of the enzyme, its physical and chemical activity parameters and its ability of surface attachment [9]. Since the new applications of laccases expose it to environments of suboptimal pH and temperature, modifications to develop more resistant enzyme must be found [54].

In the present study, we investigated several techniques that potentially may have altered laccase stability in broad ranges of pH and temperatures, obtained by chemical modifications of enzyme molecule through cross linking, deglicosylation, hydrophobization, or hydrophilization [13, 30, 41, 54]. We have also determined laccase’s resistance to halides as those factors remain a bottleneck for many new industrial applications of enzymes.

## Materials and Methods

### Medium and Growth Conditions

*C. unicolor* C-139 was obtained from the culture collection of the Regensburg University and deposited in the fungal collection at the Department of Biochemistry (Maria Curie-Sklodowska University, Poland) under the strain number 139. The crude laccase was obtained by fermentor scale cultivation in optimized Lindenberg and Holm medium [17]. The after-culture liquid was centrifuged at 10,000×*g* for 15 min, concentrated 10 times on the ultrafiltration system Pellicon 2 Mini holder (Millipore, Bedford, MA) with an Biomax 10 membrane (10 kDa cut off) and used as the source of crude enzyme.

### Enzyme Purification

The purification procedure was performed on a chromatographic EconoSystem (Bio-Rad, Richmond, VA). The semi-pure laccase was obtained after the chromatography on a DEAE-Sepharose (fast flow). The purification of laccase isoforms to homogeneity was performed using DEAE-Sepharose ion exchange, vanillyl-CPG (affinity chromatography), and chromatofocusing [45].

### Determination of Carbohydrate Content

The hydrolysis of laccase carbohydrate compounds was performed according to Niku-Paavola et al. with some modifications [39]. The 450 μl of samples (0.2 mg protein) was mixed with 50 μl 10 % SDS at 100 °C for 5 min. Then, Triton X-100 (50 μl) and N-glucosidase F (10 μl) (Calbiochem, San Diego, CA, USA) were added and incubated for 48 h at 37 °C. The obtained hydrolysates were next purified from the residual protein by ultrafiltration on Amicon Ultra-2 filter (3 kDa cut off membrane) using 10,000×*g* and analyzed by HPLC method on a VP chromatographic system (Shimadzu, Tokio, Japan) composed of a LC-10AD pump, a RID-10A refractive index detector, a SCL-10A controller, a CTO 10-AS oven (all of which were controlled by Class VP 5.03 Workstation Software; Shimadzu, 1999) and sampling valve Model 7725 (Rheodyne, Berkeley, USA) with a 20-μl loop. The mobile phase (a mixture of acetonitrile and water in the ratio 72: 28 *v*/*v*) was run at a flow rate of 1 ml/min through Kromosil–NH_2_ column (0.4 × 25 cm; 10 μm; Phenomenex) at 25 °C. The calibration of the column was carried out using the sets of sugar and sugar alcohol standards for chromatography A and B (Merck, Darmstadt, Germany).

The glucuronic acid and N-acetylglucosamine were quantified by the same HPLC system on Rezex ROA–org acid column (8 μm, 300 × 7.8 mm; Phenomenex). The mobile phase was 0.005 M H_2_SO_4_ (in Milli Q water) and the run was performed at a flow rate of 0.5 ml/min at 55 °C for the uronic acids, and 1 % phosphoric acid (in Milli Q water) at 0.6 ml/min at 25 °C for N-acetylglucosamines, respectively.

### Laccase Modification

#### Hydrophobization and Hydrophilization

The hydrophobic laccase was prepared by interactions of the -NH_2_ groups of the lysine on the enzyme's surface with palmitic acid N-hydroxysuccinimide ester (N-HSP) [37]. The aliquot of *C. unicolor* semi-pure laccase (7 mg/ml) was dissolved in 0.2 M phosphate buffer (pH 8) containing 0.2 % sodium taurodeoxycholate hydrate (NaTDC). Then, 0.5 ml of N-HSP (Sigma, St. Louis, USA) in dioxane (107 mg/ml) was added to the solution of the enzyme and allowed to react for 10 h (approximately 0.05 ml each hour) under constant rotating at 4 °C.

The hydrophilic laccase was prepared by interactions of −NH_2_ groups of the lysine on the enzyme’s surface with mono and disaccharides [8, 42]. The aliquot of *C. unicolor* semi-pure laccase (7 mg/ml) was dissolved in 0.2 M phosphate buffer (pH 8) containing 0.05 ml of 0.25 M: glucose, galactose, cellobiose, or lactose, respectively, and incubated for 24 h under constant rotating at 25 °C. The carbon-nitrogen double bonds formed in the imine were hydrogenated by addition of NaBH_4_ (5 mg/ml; 100 μl). In the modification of experiments, semi-pure laccase was preincubated with 1 mM 3,4-dimethoxybenzoic acid (Fluka, Buchs, Switzerland) in 0.1 M phosphate buffer (pH 6.5) for 1 h at 24 °C before the main reaction with N-HSP (hydrophobization) or carbohydrates (hydrophilization) was performed.

Purification of the modified enzyme was carried out using gel-filtration chromatography on a Sephadex G-25 column (Pharmacia, Uppsala, Sweden). Before chromatography, the laccase preparation was filtered through a Millex-GV membrane (Millipore) with a pore size of 0.22 μm.

Protein concentration was determined by the Bradford protein assay using bovine serum albumin as a standard [3, 25] (Bio-Rad, Hercules, CA).

#### Polymerization Assays

Three strategies of *C. unicolor* semi-pure laccase polymerization were applied. In procedure 1, the primary amine groups of laccase were first activated and cross-linked with glutaraldehyde (5 % in 0.1 M phosphoric buffer, pH 7.0) during 8 h rotation (Neolab, Heidelberg, Germany) at 10 rpm in a room temperature [37]. In procedure 2, the polymerization was performed using 1,1′-carbonyldiimidazole (CDI; Fluka-AG, Switzerland) [21] in 0.1 M McIlvaine buffer pH 4.8 and in the procedure 3 by glutaraldehyde (5 %) and carbodiimide [10].

The reaction mixtures were next purified from non reacted glutaraldehyde, carbodiimide and glutaraldehyde/carbodiimide by ultrafiltration on Amicon Ultra-2 filter (10 kDa cut off membrane) at 10,000×*g*. The volume of the concentrated sample was than diluted to the original volume and centrifuged at 10,000×*g*. This procedure was performed three times in order to obtain pure agglomerates of laccase.

The semi-pure laccase samples with inactivated catalytic site were prepared by addition of 1 mM 3,4-dimethoxybenzoic acid (Fluka, Buchs, Switzerland) in 0.1 M phosphate buffer (pH 6.5) and incubated for 1 h at 24 °C before proceeding to the polymerization assay.

### Detection of Lysine Groups

The number of covalently modified amino groups of the laccase Lys residues was determined by the procedure based on the interaction of 2,4,6-trinitrobenzenesulfonic acid (TNBS) with the nonmodified Lys residues of the enzyme [8, 41]. Aqueous solution of 4 % NaHCO_3_ and 0.1 % TNBS were added to an aqueous solution of the laccase (2.5–5.0 mg) in 2 % SDS. The reaction mixture was incubated in the dark at 40 °C for 2 h. A 10 % SDS solution (0.5 ml) and 1 M HCl (0.5 ml) were added into each tube to terminate reaction. The number of free amino groups was evaluated using a standard curve prepared for leucine (OD = 335 nm).

### Laccase Activity Assay

Absolute enzymatic yield of laccase activity in the liquid cultures was measured spectrophotometrically at 525 nm with the use of UV–Vis 160 A Shimadzu spectrophotometer (Tokyo, Japan) [14, 25] using syringaldazine as a substrate. Enzyme and substrate blanks were included. One unit (nano katal, nkat) of laccase activity was defined as the amount of the enzyme catalysing the production of one nanomole of colored product (quinone, ε^M^ = 65,000 M^-1^ cm^-1^) per second at 25 °C and pH 7.4. The activity was expressed as nano katals per liter of culture medium (nkat/L). The protein concentration was measured by Bradford protein assay using bovine serum albumin as a standard [3]. A Zetasizer 3000 (Malvern, UK) instrument was used to measure zeta potential and size of protein aggregates in tested samples. Samples prepared for the dynamic light scattering (DSL) measurements were loaded into a pre-rinsed folded capillary cell. The particle size was taken as a mean value of five measurements.

### Effect of Temperature and pH on Laccase Activity

Laccase activity in native and modified samples was assayed at different ranges of temperatures (20–90 °C) and pH (3.0–8.0) at 20 °C. Samples were prepared in 0.1 M McIlvaine buffer.

### Effect of Halides Ions on Laccase Activity

The effect of ionic strength of halide ions on native and modified laccase activity was measured by addition of NaF, NaCl, KI, and NaF (all 0.1 M) to the samples dissolved in 0.2 M McIlvaine buffer (pH 5.3) at concentration ranges 0–0.2 M at 20 °C. The reversibility of inhibition by halides was estimated after 1 and 24 h with and without ascorbate [4], using size exclusion chromatography on Sephadex G-25 column (25 × 1.5 cm) stabilized by MilliQ water.

## Results and Discussion

In this study, we demonstrated that the production of laccase was considerably enhanced by the addition of micromolar concentrations of Cu^2+^ into carbon and nitrogen-sufficient medium (C/N = 16.69). The fermentor scale cultivation of *C. unicolor* resulted in higher production of crude laccase than observed in submerged cultures similarly to Janusz et al. [18]. Obtained enzyme was purified to homogeneity by rapid procedure using a combination of ion-exchange chromatography, affinity chromatography, and chromatofocusing. The partial physical, chemical and kinetic characterization (MW, p*I*, total carbohydrate contents, *K*_m_, *V*_max_) of laccase isoforms from *C. unicolor* have been previously described [44, 45]. Since tested isoforms differed in their carbohydrate content (1.6–3.5 %) as well as in substrate specificity, analysis of their carbohydrate composition was performed (Table 1). Structurally, glycoproteins consist of a polypeptides covalently bond to a carbohydrate moiety [45]. The saccharide residuals can be linked to a polypeptide chain in two different ways. First are commonly found O-glycosidic linkages that involve attachment of the carbohydrate to the hydroxyl group of serine, threonine or hydroxylisine and the N-glycosidic linkages that involve attachment to the amide group of asparagine [52]. The data collected indicated that all investigated glycoproteins contained up to 50 % of mannose as well as other monosaccharides such as: fucose, galactose, N-acetyl-glycosamine, N-acetyl-galactosamine, glucuronic acid, galactouronic acid, and sialic acid residues. Those findings were in agreement with previously reported results [6, 11, 24] and suggested that in all four *C. unicolor* laccase isozymes carbohydrate residues were attached via N-glycosidic linkages to the asparagine. It is interesting that N-acetyl-galactosamine was present in the Lac II isoform instead of N-acetyl-galactosamine form that is the most commonly distributed among other laccase isoforms [6, 11, 24].

**Table 1:** Carbohydrate content of isoforms from laccase of *C. unicolor*

Carbohydrate		Cerrena unicolor isoform			
		Lac Ia1	Lac Ia2	Lac Ib	Lac II
Carbohydrate content of N-linked sugars [mol%]	Mannose	59.36	58.41	63.33	57.26
	N-acetyl-glucosamine	38.20	34.63	36.47	–
	N-acetyl-galactosamine	–	–	–	35.59
	Glucose	–	–	–	–
	Galactose	–	1.11	–	–
	Glucuronic acid	0.42	–	0.09	–
	Galacturonic acid	–	–	0.11	–
	Sialic acid	1.26	1.70	–	–
	Fucose	0.76	4.15	–	7.15
	Arabinose	–	–	–	–

As previously indicated [40], −NH_2_ groups of lysine residues are the most preferred sites for enzyme's surface modifications. It is generally believed that the introduction of hydrophilic groups onto the surface of a protein may improve its stability and form additional electrostatic interactions, hydrogen bonds or salt bridges [37]. On the other hand, addition of hydrophobic groups may also contribute to protein stabilization and its activity [22]. Here, the number of Lys residues in semi-pure *C. unicolor* laccase was determined using TNBS method. For samples with four isoforms, ten lysine residues were detected. This number was consistent with the results obtained from *C. unicolor* lac Ia1 isozyme gen and cDNA characterization [16]. Predicted protein sequence of *lac Ia1* isoform dominated (27 %) in semi-pure laccase samples and contained 11 lysine residues. After modification of laccase with the fatty acid esters of N-hydroxysuccinimide three unmodified lysine residues were detected. In the case of samples preincubated with veratric acid (structural substrate analog) that inactivates enzyme catalytic site, five lysine residues were observed. This finding may be indicative of presence of two Lys residues in laccase catalytic center. Studies of known *Cerrena spp.* laccase protein sequences (predicted using known cDNA sequences) revealed that its molecule contains 4, 7, 9, or 11 Lys residues (ACZ58367, ACZ58368, ACZ58369, 3DIV_A, AEQ35306). It can be estimated that in our experiments five exposed Lys residues were located on the surface of laccase molecule. The covalent attachment of hydrophobic groups to the enzyme could lead to the formation of a polymeric surfactant-like molecule, which may undergo spontaneous aggregation. Measured mean size of particles distribution for *C. unicolor* native laccase (semi-pure) was 100 nm, and for hydrophobized one 1,000 nm. Whereas, hydrophobized sample in the presence of veratric acid had the mean diameter of 300 nm, which could be indicative of its clustered structure. All modified laccase solutions exhibited higher negative potentials (−34 and −20 mV) in comparison to the native ones (−12 mV). It can be speculated, that the differences in potentials came from the lower number of free Lys residues in the case of modified samples, thus there was an observed decrease in the number of positively charged moieties while the number of negatively charged residues did not change [35].

**Fig. 1:**
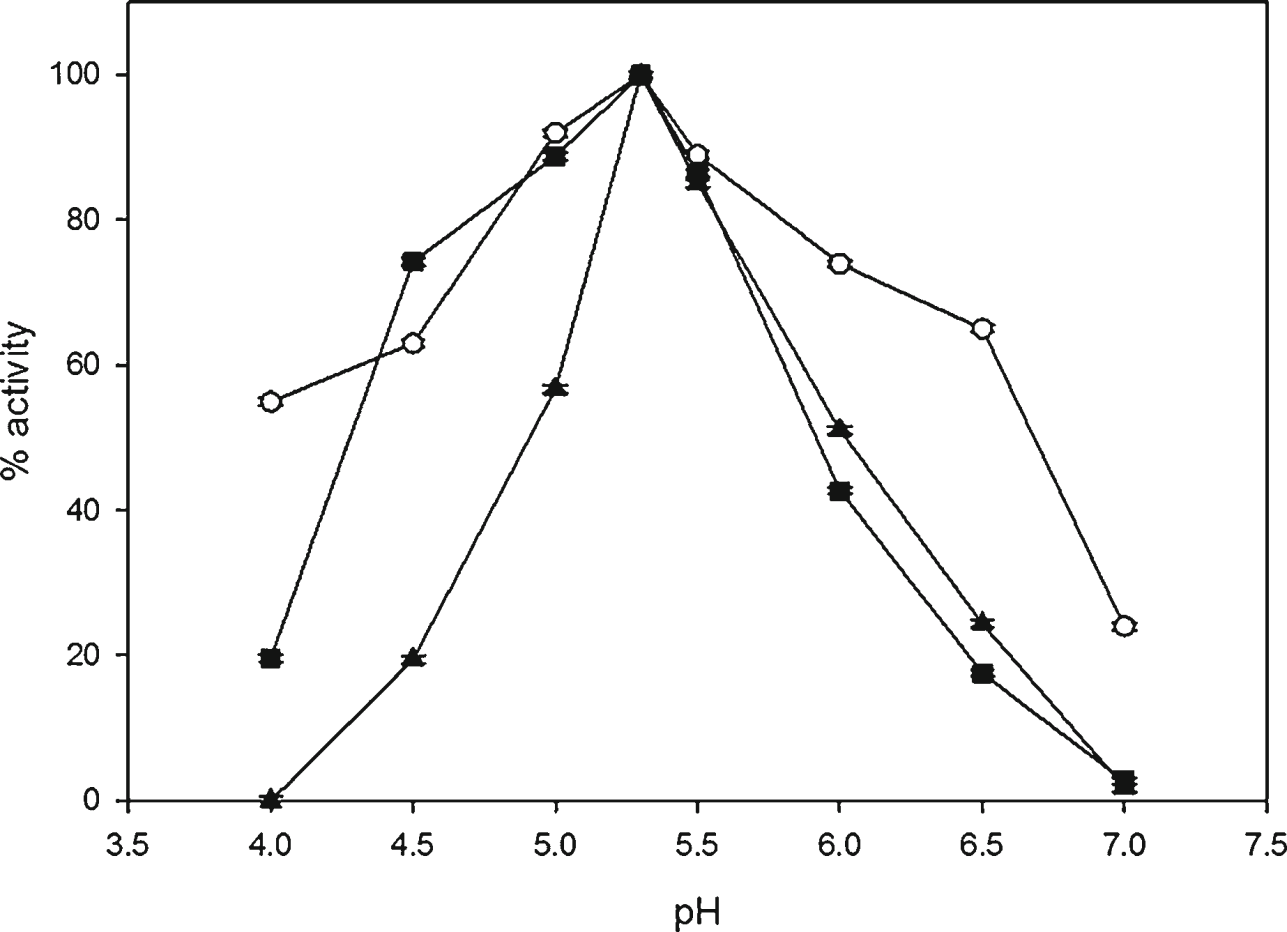
*C. unicolor* laccase activity dependence on pH for solutions of: *circle* native, *filled triangle* hydrophobized, and *filled square* hydrophobized with inactivated catalytic site coated with 3,4-dimethoxybenzoic acid. Error bars are ±1 SD. Where error bars cannot be seen, they fall within the data symbol

**Fig. 2:**
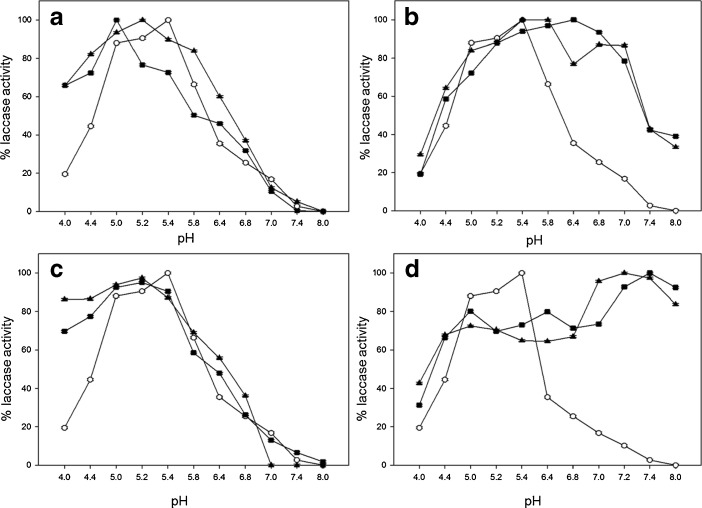
*C. unicolor* laccase activity dependence on pH for solutions of: circle native, *filled triangle* hydrophilized, and *filled square* hydrophilized with active site coated with 3,4-dimethoxybenzoic acid. Modifications of enzyme were performed with: **a** glucose, **b** galactose, **c** lactose, and **d** cellobiose. *Error bars* are ±1 SD. Where error bars cannot be seen, they fall within the data symboll

Further experimental analysis of native and modified laccase solutions after hydrophobization with palmitic acid, with and without 3,4-dimethoxybenzoic acid, uncovered significant decreases in enzymatic activities by 18 % and 39 %, respectively, but showed the same optimal pH for laccase after hydrophobization (Fig. 1). In contrast after hydrophilization with glucose (Fig. 2a) or cellobiose (Fig. 2d) the pH optimum shifted to less acidic values, whereas using galactose (Fig. 2b) and lactose (Fig. 2c) gave reversed effect. Modifications with glucose and 3,4-dimethoxybenzoic acid led to the shift in the optimum pH from 5.3 to 6.8 and from 5.3 to 5.8 for the laccase solutions with glucose only (Fig. 2a). Hydrophilization with galactose (Fig. 2b) moved pH optimum towards more acidic values (5.2), while with galactose and 3,4-dimethoxybenzoic acid to pH 5.0.

**Fig. 3:**
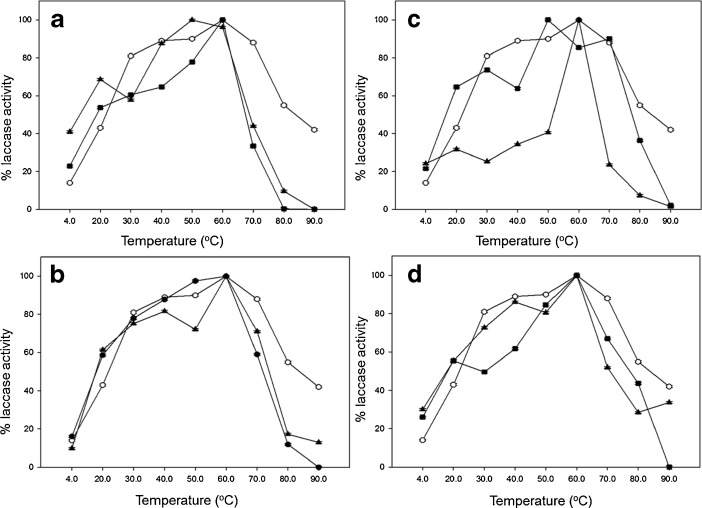
*C. unicolor* laccase activity dependence on temperature for solutions of: *circle* native, *filled triangle* hydrophilized, and *filled square* hydrophilized with active site coated with 3,4-dimethoxybenzoic acid. Modifications of enzyme were performed with: **a** glucose, **b** galactose, **c** lactose, and **d** cellobiose. *Error bars* are ±1 SD. Where error bars cannot be seen, they fall within the data symbol

Moreover, optimal temperatures tested for laccase solutions varied depending on modification. In the case of samples modified with galactose (Fig. 3b) and cellobiose (Fig. 3d), optimum temperatures were reported at 60 °C, whereas with glucose (Fig. 3a) and lactose (Fig. 3c) were observed at the value of 50 °C. The increase in thermal stability by immobilizing modified laccase solutions to glutaraldehyde-activated matrices, with the establishment of multiple Schiff-base linkages between free amino groups in the protein and the aldehyde group in the glutaraldehyde linker, are often observed [43].

The copolymerization of tested laccase solutions resulted in higher durability of their activity on the surface unit [26] and relatively higher potential, which may be significant for its future usage in construction of electrochemical cathodes [36].

**Fig. 4:**
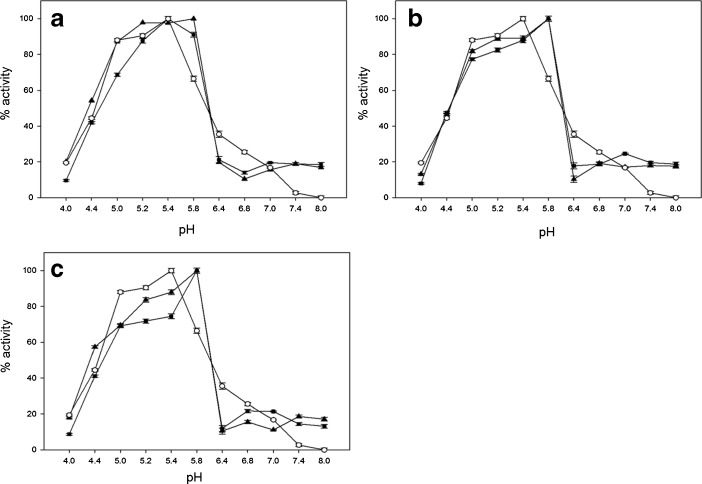
*C.unicolor* laccase activity dependence on pH for solutions of: *circle* native, *triangle* copolymerized, and *square* copolymerized with active site coated with 3,4-dimethoxybenzoic acid. Modifications of enzyme were performed with **a** glutaraldehyde, **b** CDI, and **c** glutaraldehyde and CDI. *Error bars* are ±1 SD. Where error bars cannot be seen, they fall within the data symbol

To date, the most popular homobifunctional reagents for protein immobilization or copolymerization are: glutaraldehyde, diisocyanates and diisothiocyanates, homopolyfunctional epoxides; “zero-length crosslinking” with the use of carbodiimides (CDI), acyl azide, or dye-mediated photo-oxidation [28, 31, 46, 47]. For this reason, copolymerization of laccase was performed with the use of glutaraldehyde and carbodiimide (Fig. 4). Here, decreases in pH (∼0.5 unit) towards less acidic values were determined for samples modified by glutaraldehyde, CDI, and samples with catalytic site inactivated by 3,4-dimetoxybenzoic acid.

**Fig. 5:**
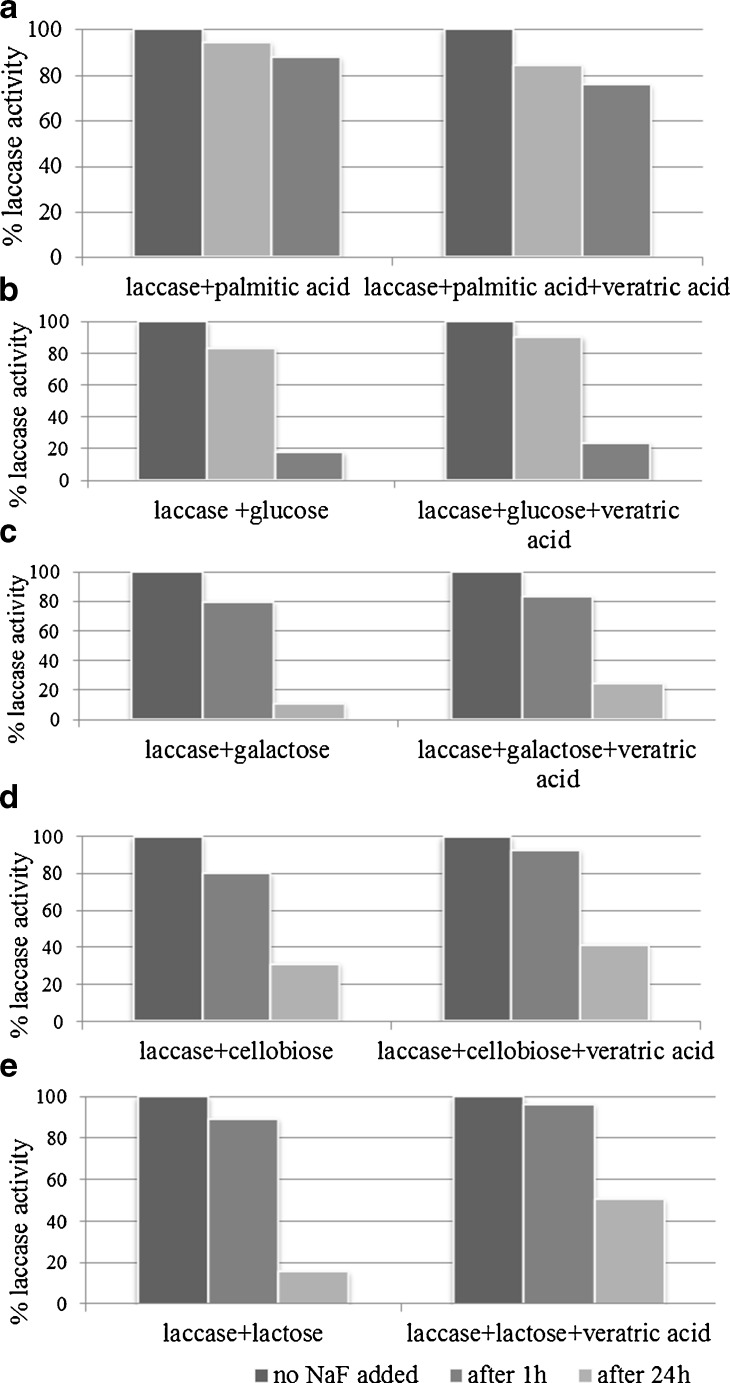
Reversibility of *Cerrena unicolor* laccase inhibition by F^−^ ions after 1 and 24 h preincubation. The modification of laccase were performed using: **a** palmitic acid, **b** glucose, **c** galactose, **d** cellobiose, and **e** lactose

**Fig. 6:**
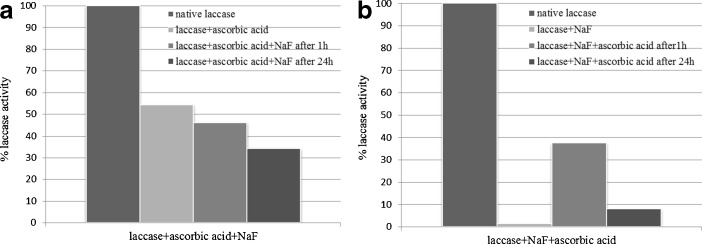
The reversibility of native *Cerrena unicolor* laccase inhibition by F^−^ ions after 1 and 24 h preincubation in the presence of ascorbic acid. The reduction was performed: **a** before and **b** after addition of fluoride ions

It has been previously demonstrated that the presence of halogenated compounds can be a drawback when using laccase in biotechnological processes since halides (especially fluorides and chlorides) inhibit enzyme’s catalytic activities [1, 49]. The inhibitory effect of the halides (F^−^ > Cl^−^ > J^−^) seemed to decrease with an increase in its size or a decrease in its electric potential. Results with the use of agglomerated molecules of laccase treated with halides (Table 2) showed that all of the altered enzyme molecules exhibited higher stability in the presence of tested ions, as previously described by Branden et al. [4]. During the treatments with halides (concentrations of 0–0.2 M) laccase samples exhibited the fastest inhibition of catalytic activities with fluoride ions, although samples modified with lactose (with and without addition of 3,4-dimethoxybenzoic acid) had lower sensitivity towards F^-^ and lower decrease in catalytic activity in its presence. This phenomenon may be attributed to the formation of stable coating around enzyme molecule by tested mono and disaccharides and postponing fluoride diffusion to the catalytic center T2/T3. This discovery was also in agreement with the fact that fluoride has an ability to block thiol groups in free −SH of amino acids at central positions of enzyme active sites, thus making the formation of S–S bridges impossible. Fluoride can also form hydrogen bonds with amides that are stronger than −N–H–O– hydrogen bonds. This would be of paramount importance for the formation and maintenance of steric structure of proteins in the catalytic centers [29]. As discussed before, the affinity of laccase for F^−^ was so strong that the enzyme as usually prepared was contaminated, to 65 %, of the molecules in a given preparation having this inhibitor bound to Type 2 Cu^2+^ [49, 52]. It has not been possible to remove the inhibitor completely by extensive dialysis [7], however, dialysis in the presence of ascorbate, which kept the enzyme in the fully reduced form, led to a better removal of halides (also F^−^ ions). Here, in all tested cases, the size exclusion method led to an average 80 % inhibition removal after an hour (Fig. 5). Samples hydrophilized with lactose and cellobiose (and with inactivated catalytic site) showed an additional 90 % reversibility after an hour of finalizing the reaction and 40 % after 24 h. In both cases, laccase hydrophobized with palmitic acid (Fig. 5a) showed much higher reversibility levels after 1 (above 80 %) and 24 h (above 70 %) incubation with fluoride ions. The presence of ascorbate added to laccase solutions before F^−^ ions (Fig. 6a) resulted in higher reversibilities in comparison to the conditions where reagents were used in the reversed order (Fig. 6b).

**Table 2:** Effect of halides on laccase activity

Modificators		Laccase sample activity [%]										
Halogenated compound	Concentration [M]	Native	Hydrophobized		glucose		Galactose		cellobiose		lactose	
				+veratric acid		+veratric acid		+veratric acid		+veratric acid		+veratric acid
	0.00	100										
NaF	0.01	12.93	10.82	7.12	85.44	92.68	83.87	87.46	27.15	45.93	74.71	81.28
	0.02	5.79	4.40	2.67	70.90	79.57	64.86	79.62	22.69	44.30	60.11	65.78
	0.0.3	2.57	2.74	1.14	56.35	65.31	53.31	63.34	16.54	41.06	48.87	53.48
	0.04	1.17	2.20	0.76	39.19	58.32	45.36	50.60	14.17	37.68	41.01	44.92
	0.05	1.09	1.61	0.38	23.76	32.79	34.57	42.05	5.85	31.39	29.77	34.76
	0.06	0.7	0.73	0.16	18.21	27.52	25.61	28.85	5.33	27.69	23.03	26.74
	0.07	0.00	0.47	0.00	17.58	20.57	19.32	25.45	3.69	22.77	14.04	18.72
	0.08	0.00	0.00	0.00	12.42	17.79	18.13	21.37	3.23.	13.40	9.55	13.90
	0.09	0.00	0.00	0.00	10.58	15.23	6.98	11.85	1.87	11.45	6.18	12.83
	0.10	0.00	0.00	0.00	4.13	11.83	5.13	7.13	1.00	10.30	4.49	10.16
	0.20	0.00	0.00	0.00	1.75	3.75	0.09	0.78	0.00	1.79	3.37	6.95
NaCl	0.01	48.49	45.61	42.24	97.78	98.31	72.58	41.04	51.32	41.04	84.44	87.89
	0.02	29..34	26.47	27.41	88.18	91.48	46.77	22.48	35.75	22.48	78.16	72.66
	0.03	12.57	17.47	13.82	56.75	65.39	20.62	12.89	31.23	12.89	52.56	65.92
	0.04	6.14	8.65	6.46	45.22	52.15	43.53	5.47	19.82	5.47	44.22	59.55
	0.05	5.20	4.94	4.76	32.29	33.29	6.55	2.46	23.39	2.46	30.39	33.05
	0.06	2.67	2008	2.21	21.21	22.58	6.03	1.11	16.87	1.11	8.23	22.29
	0.07	1.78	0.42	0.85	5.93	17.24	4.77	0.73	9.52	0.73	3.67	6.48
	0.08	0.27	.20	.25	4.84	9.79	4.66	0.27	2.19	0.27	2.48	5.10
	0.09	0.15	0.11	0.13	2.90	7.38	4.34	0.15	1.20	0.15	1.44	3.67
	0.10	0.03	0.02	0.04	2.92	20.08	2.40	0.30	0.18	0.30	0.98	2.86
	0.20	0.00	0.00	0.00	1.69	0.00	1.37	0.00	0.23	0.00	0.32	0.32
NaJ	0.01	95.28	78.00	79.56	7.94	83.60	95.29	95.01	92.29	96.46	97.90	99.85
	0.02	89.35	60.31	54.23	60.51	65.36	62.48	68.20	76.51	86.20	76.52	80.35
	0.03	77.91	45.51	37.08	53.90	59.32	53.16	54.04	75.25	65.93	27.43	48.33
	0.04	63.47	35.56	32.07	39.72	44.20	43.64	51.76	62.37	61.44	11.03	29.05
	0.05	53.76	23.69	16.44	30.50	35.14	42.32	44.03	48.11	53.99	10.02	22.17
	0.06	22.89	11.89	10.85	24.82	28.34	31.67	35.81	45.95	51.69	4.70	19.06
	0.07	20.71	8.45	3.67	7.12	17.09	4.19	5.29	37.27	44.37	3.11	11.02
	0.08	18.16	4.20	2.13	6.82	11.80	2.21	2.45	21.45	37.36	1.82	9.67
	0.09	15.89	2.31	1.46	5.90	7.27	1.12	1.66	14.51	22.16	1.27	7.44
	0.10	12.58	1.02	0.90	0.74	3.37	0.68	0.97	5.19	11.59	1.22	5.90
	0.20	10.26	0.56	0.00	0.00	0.88	0.07	0.43	0.35	2.16	0.05	0.48

## Conclusions

The primary task of this work was to perform chemical modifications of laccase from *C. unicolor* in order to increase stability of the enzyme in broad ranges of pH and temperatures. From the beginning, the catalytic activity and nativity of the enzyme solutions were confirmed using spectral and electrochemical methods. Hydrophobization of the laccase was achieved and resulted in its increased resistance to halides. During the treatments with various halides, fluoride was the strongest inhibitor of laccase catalytic activities. However, as showed here, samples modified by hydrophilization had lower sensitivity towards F^−^ and a smaller decrease in catalytic activity in its presence. Modifications by hydrophobization have also attributed to a higher reversibility of fluoride inhibitory effect after 24 h of preincubation as well as in the presence of the ascorbate solution.
